# Falls efficacy instruments for community-dwelling older adults: a COSMIN-based systematic review

**DOI:** 10.1186/s12877-020-01960-7

**Published:** 2021-01-07

**Authors:** Shawn Leng-Hsien Soh, Judith Lane, Tianma Xu, Nigel Gleeson, Chee Wee Tan

**Affiliations:** 1 Singapore Institute of Technology, Singapore, Singapore; 2 Queen Margaret University, Musselburgh, UK; 3 Glasgow Caledonian University, Glasgow, UK

**Keywords:** Falls efficacy, Balance confidence, Older adults, Psychometric, COSMIN

## Abstract

**Background:**

Falls efficacy is a widely-studied latent construct in community-dwelling older adults. Various self-reported instruments have been used to measure falls efficacy. In order to be informed of the choice of the best measurement instrument for a specific purpose, empirical evidence of the development and measurement properties of falls efficacy related instruments is needed.

**Methods:**

The Consensus-based Standards for the Selection of Health Measurement Intruments (COSMIN) checklist was used to summarise evidence on the development, content validity, and structural validity of instruments measuring falls efficacy in community-dwelling older adults. Databases including MEDLINE, Web of Science, PsychINFO, SCOPUS, CINAHL were searched (May 2019). Records on the development of instruments and studies assessing content validity or structural validity of falls efficacy related scales were included. COSMIN methodology was used to guide the review of eligible studies and in the assessment of their methodological quality. Evidence of content validity: relevance, comprehensiveness and comprehensibility and unidimensionality for structural validity were synthesised. A modified GRADE approach was applied to evidence synthesis.

**Results:**

Thirty-five studies, of which 18 instruments had been identified, were included in the review. High-quality evidence showed that the Modified Falls Efficacy Scale (FES)-13 items (MFES-13) has sufficient relevance, yet insufficient comprehensiveness for measuring falls efficacy. Moderate quality evidence supported that the FES-10 has sufficient relevance, and MFES-14 has sufficient comprehensibility. Activities-specific Balance Confidence (ABC) Scale–Simplified (ABC-15) has sufficient relevance in measuring balance confidence supported by moderate-quality evidence. Low to very low-quality evidence underpinned the content validity of other instruments. High-quality evidence supported sufficient unidimensionality for eight instruments (FES-10, MFES-14, ABC-6, ABC-15, ABC-16, Iconographical FES (Icon-FES), FES–International (FES-I) and Perceived Ability to Prevent and Manage Fall Risks (PAPMFR)).

**Conclusion:**

Content validity of instruments to measure falls efficacy is understudied. Structural validity is sufficient for a number of widely-used instruments. Measuring balance confidence is a subset of falls efficacy. Further work is needed to investigate a broader construct for falls efficacy.

**Supplementary Information:**

The online version contains supplementary material available at 10.1186/s12877-020-01960-7.

## Background

Escalating consumption of healthcare services globally, associated with high rates of falls-related morbidity in rapidly ageing demographics, has become a major public health concern among policymakers, researchers and clinicians [1–3]. Falls efficacy can be better addressed among older adults to maximise their independence, promote maintenance of an active lifestyle and counter burdensome associations [4]. Falls efficacy as a latent construct in community-dwelling older adults has been widely studied in research and clinical practice [5]. Conceptualised using Bandura’s self-efficacy theory [6], the assessment of falls-related self-efficacy conventionally focuses on beliefs and confidence about one’s ability to undertake activities of daily living without falling [7]. Over the last three decades, falls efficacy has been studied alongside other falls-related psychological constructs, i.e. fear of falling and balance confidence [8]. Commonly-used self-reported instruments used to measure falls efficacy include the Falls Efficacy Scale (FES) [7], Modified Falls Efficacy Scale (MFES) [9], Activities-specific Balance Confidence Scale (ABC) [10], CONFBal scale of balance confidence (CONFBal) [11], Falls Efficacy Scale-International (FES-I) [12] and Iconographical Falls Efficacy Scale (Icon-FES) [13]. Selecting appropriate instruments to measure falls efficacy is obscured by operational heterogeneity amongst relevant psychological constructs such as fear of falling and balance confidence [8]. High-quality psychometric evidence should underpin the selection of specific instruments.

Researchers and clinicians have used different instruments to measure falls-related psychological constructs interchangeably. The first of such scale, FES [7], was developed in 1990. The FES, underpinned by established theoretical models of cognitive process underlying emotions, had been used to measure fear of falling, i.e. low falls efficacy scores to indicate high fear of falling in older adults [7]. However, this conflation of related or mediating but essentially distinct theoretical constructs has been criticised. Falls efficacy may be used to mediate the relationship between fear of falling and falls [14]. Further, falls efficacy and fear of falling can be influenced differently by other psychological concepts, including depression [15]. Expansive assessment scales with good psychometric properties, i.e. The Survey of Activities and Fear of Falling in the Elderly (SAFE) [16], The University of Illinois at Chicago Fear of Falling Measure (UICFFM) [17] and the Geriatric Fear of Falling Measure (GFFM) [18] may facilitate a broader understanding of the fear of falling amongst other emotional (e.g. anxiety) and behavioural (e.g. activity avoidance) psychological elements. Since the mid-1990s, other instruments have been further developed to address the FES’s varied limitations, including the ABC [10], which had been shown to be highly correlated to the FES (.86) [19]. The ABC was conceptualised to measure balance confidence within broad-ranging assessments of functional activities. The abbreviated version of the balance confidence measure, ABC-6 [20], was developed from patient groups with Parkinson’s disease and high-level gait disorders who reported highest level of fear in their task performance. These instruments were frequently identified as measures of fear of falling and had limited clinical utility to assess balance confidence in older and frailer people who are unable to perform high-level activities [21]. By the end of the 2000s, falls efficacy instruments were advocated for measuring the latent construct of balance confidence [22]. The cue question in ABC-Simplified (ABC-S) was reworded from “How confident are you that you *will not lose* your balance or become unsteady when you …” , to, “Up to what point are you confident that you will *maintain* your balance when you do the following activities?” [23]. Another instrument, CONFbal, derived using a 21-item instrument, ‘Confidence in Everyday Activities’ [24], was used to measure an older and frailer person’s cognitive (belief) rather than emotional (fear) constructs with the intent of physiotherapy-focused rehabilitation training [11]. Some evidence, including that from systematic reviews of falls-related psychological concerns in community-dwelling older adults, suggested that assessing falls efficacy and balance confidence was tautologic due to commonality of items amongst instruments [22]. However, conflicting evidence has also challenged accepting balance confidence and falls efficacy to be isomorphic constructs. For example, a recently developed scale, Perceived Ability to Prevent and Manage Fall Risk (PAPMFR), was used to measure a wide range of fall-related perceptions and treats falls efficacy conceptually as a broad entity [4].

Previous efforts were made to recommend ‘gold standard’ instruments for specific falls-related psychological constructs for clinical use in two antecedent systematic review. Jostad et al. [25] presented key measurement properties of the different instruments, including details of the populations in which measures have been tested, as well as information on scaling, to aid researchers and clinicians with their selection of an instrument. Moore et al. [8] focused attention on the psychometric properties of common instruments used in independent-living and community-dwelling older adults and recommended that MFES, FES-I and ABC could be used to measure falls efficacy and balance confidence. However, neither antecedent review was able to offer a critical evaluation of each instrument’s content validity, empirical evidence to justify its use, and hence, the inherent quality of the evidence. Content validity, which refers to “the degree to which an instrument measures the construct it purports to measure”, would provide empirical evidence to justify the use of appropriate instruments [26]. Countering this fundamental gap in the literature could lead to facilitating confidence among researchers and clinicians in their selection of instruments to measure falls efficacy.

The Consensus-based Standards for the Selection of Health Measurement Instruments (COSMIN) methodology facilitates systematic review of measurement instruments. It offers a hierarchical psychometric process by which any endorsed instrument would have needed to satisfy priority and bias-free evidential thresholds of both content and structural validity (i.e. scores of an instrument adequately reflect the dimensionality of the construct to be measured) [27]. Thus, transparent and evidence-based recommendations can be made for the selection of appropriate instruments to measure intended constructs [28]. To the best of the authors’ knowledge, there has not been any systematic reviews that had adopted the COSMIN methodology to evaluate falls efficacy-related instruments. The purpose of this paper is to systematically review content and structural validity of falls efficacy-related scales for community-dwelling older adults, using COSMIN guidelines.

## Methods

### Protocol and registration

This review was conducted in accordance with the Preferred Reporting Items of Systematic Reviews and Meta-Analyses Protocol (PRISMA) guidelines [29] (Additional file 8). A protocol for this systematic review was registered in PROSPERO (Ref-CRD42019124366).

### Eligibility criteria

Studies were included if instruments measuring constructs relating to ‘falls-efficacy’, ‘falls-related self-efficacy’ and ‘balance confidence’ in community-dwelling older adults, including translated and culturally adapted versions. Development studies of falls efficacy instruments that interpreted fear of falling were included because of the convolved history. However, studies were excluded if titles were related specifically to, and measured constructs such as ‘fear’, ‘anxiety’ as well as ‘activity avoidance’.

### Search strategy and selection criteria

A comprehensive language-unrestricted search was conducted between 1st January 1990 and 31st May 2019 amongst Medline (EBSCOhost), Web of Science Core Collection, PsychInfo (EBSCOhost), Scopus (scopus.com) and Cinahl Plus with full text (EBSCOhost) databases. COSMIN-guided searching consisted of three groups of search terms using Boolean operators, detailing: (1) construct of interest, (2) target population and (3) measurement properties (see Additional file 1).

Studies that focused on the development of falls efficacy related instruments measuring falls efficacy or balance confidence were included for the assessment of content validity (Table 1). Content validity studies were eligible if they were full-text original articles that featured community-dwelling older adults or professionals (e.g. falls-related researchers, clinicians), in order to assess the relevance, comprehensiveness, or comprehensibility of the content of at least one instrument. Cross-cultural adaptation studies of instruments were included, if comprehensibility pretesting of the adapted questionnaire within the target population had been performed. Similarly, the availability of content validity studies for instruments in comparable populations were included. Structural validity studies were included only as full-text original articles about community-dwelling older adults, assessing instrument dimensionality via factor or item response theory analysis [30].

**Table 1:** Instruments measuring falls-related self-efficacy or balance confidence

Instrument (Abbreviation)	Recall period	Number of items	Response options	Total score range	Interpretation of results
**List of falls efficacy scales**					
Falls Efficacy Scale - 10 items (FES-10)	Undefined	10	1–10	10–100	Higher score indicate lower efficacy
Modified Falls Efficacy Scale - 11 items (MFES-11)	Undefined	11	1–3	11–33	Higher score indicate higher efficacy
Modified Falls Efficacy Scale - 12 items (MFES-12)	Undefined	12	1–4	12–48	Higher score indicate higher efficacy
Modified Falls Efficacy Scale - 13 items (MFES-13)	Undefined	13	0–10	0–130	Higher score indicate higher efficacy
Modified Falls Efficacy Scale - 14 items (MFES-14)	Undefined	14	0–10	0–140	Higher score indicated higher efficacy
Perceived Ability to Prevent and Manage Fall Risks (PAPMFR)	Undefined	6	1–5	6–30	*items scores were reversed-coded to represent higher scores indicate higher efficacy.
Revised Gait Efficacy Scale - 8 items (GES-8)	Undefined	8	1–10	8–80	Higher score indicate higher efficacy
Gait Efficacy Scale - 10 items (GES-10)	Undefined	10	1–10	10–100	Higher score indicate higher efficacy
Perceived Control Over Falling (PCOF)	Undefined	4	1–4	4–16	Higher score indicate higher efficacy
Perceived Ability to Manage Risk of Falls or Actual Falls (PAMF)	Undefined	5	1–4	5–20	Higher score indicate higher efficacy
Balance Self-Perceptions Test (BSPT)	Undefined	20	1–5	20–100	Higher score indicate higher efficacy
**List of balance confidence scales**					
Activities specific Balance Confidence scale – Short (ABC-6)	Undefined	6	0–100	0–600	Higher score indicate higher efficacy
Activities specific Balance Confidence scale – Simplified (ABC-15)	Undefined	15	0–3	0–45	Higher score indicate higher efficacy
Activities specific Balance Confidence scale (ABC-16)	Undefined	16	0–100	0–1600	Higher score indicate higher efficacy
CONFBal scale of balance confidence (CONFBal)	Undefined	10	1–3	10–30	Higher score indicate lower efficacy
**List of scales not measuring falls efficacy or balance confidence**					
Iconographical Falls Efficacy Scale (Icon-FES)	Undefined	30	1–4	30–120	Higher score indicating greater concerns of falling
Falls efficacy scale – International (FES-I)	Undefined	16	1–4	16–64	Higher score indicating greater concerns of falling
Mobility Efficacy Scale (MES)	Undefined	10	1–4	10–40	Higher score indicate greater concerns of falling

Two independent reviewers (SS; CWT) interrogated database-derived titles and abstracts for eligibility and subsequently, full texts for potential inclusion. Consensus was sought, but any disagreements were resolved by an additional team-based reviewer.

### Quality assessment and data extraction

The COSMIN checklist guided the assessment about methodological quality of studies detailing an instrument’s development, content validity and structural validity [28, 30]. The 35 criteria ensured the relevance of an instrument’s items and quality amongst cognitive interviewing or other piloting of comprehensibility and comprehensiveness. A further 31 criteria assessed a study’s methodological quality of content validity involving the relevance, comprehensiveness, and comprehensibility within the target population, as well as relevance and comprehensiveness amongst professional participants. Four criteria evaluated the appropriateness of the statistical methods assessing structural validity of an instrument. Criteria were characterised on 4-point rating scales, namely, “very good”, “adequate”, “doubtful” (reflecting methods that had not been described clearly) or “inadequate” (reflecting methods that had not been described); with overall ratings regulated by recording lowest rating among relevant items [30]. Ultimately, overall ratings about studies’ methodological qualities influenced the interpretation of evidential quality of the psychometric measurement property of the instrument [27].

Measurement properties of studies were evaluated via COSMIN and their distribution amongst three pairings of two reviewers (SS, CWT; SS, JL; SS, TX), with discussions determining consensus. Information extracted included the construct to be measured, target population, and context of use (instrument development studies); patient characteristics (concept elicitation and cognitive interview studies; validity studies); and results (validity studies). Data were extracted by the first reviewer who had been paired, while the second reviewer double-checked the accuracy of the extracted information.

### Evidence synthesis

The following steps were conducted to synthesise evidence by each pair of reviewers (SS, CWT; SS, JL; SS, TX). First, the results of instrument development and content validity studies were rated according to guided criteria so as to evaluate relevance, comprehensiveness and comprehensibility. Each criterion was rated as sufficient (+), insufficient (−) or indeterminate (?). Second, an overall result was obtained by pooling the results of all available studies and reviewers’ ratings on the same instrument (regardless of language and country) [30]. The studies on structural validity were rated according to a recommended criteria guide published by Prinsen and colleagues (see Additional file 2) [27]. Taking all evidence into account, the overall structural validity of the instrument was rated as sufficient (+), insufficient (−), inconsistent (±) or indeterminate (?). Third, the quality of evidence was rated according to a modified GRADE approach taking into account the study quality, consistency of results across studies and reviewers’ rating (for content validity only). The overall rating was graded for the quality of the evidence using a modified GRADE approach (high, moderate, low or very low) [27].

## Results

**Fig. 1:**
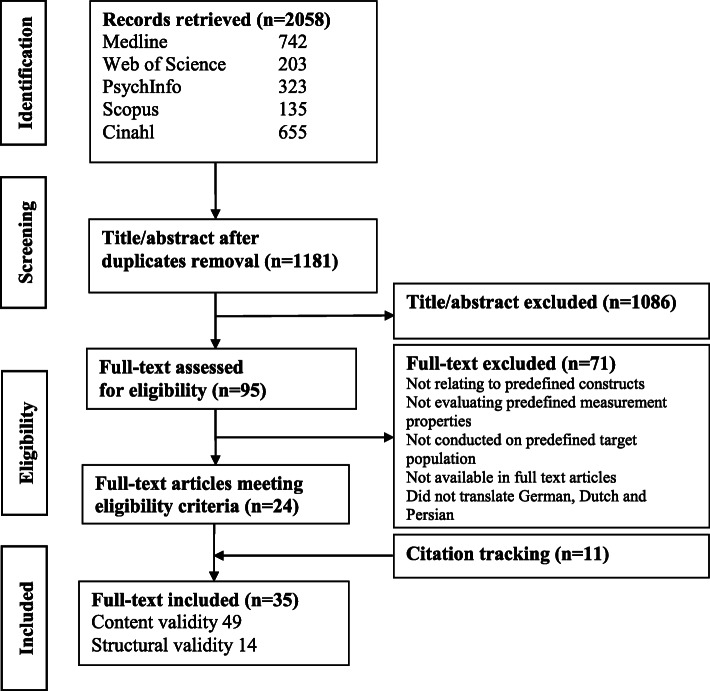
Flow chart of results of search strategy and selection of records

From an initial 2058 records, 95 were retrieved for full-text review, and 24 were selected (Fig. 1). Seventy-one records were excluded: 44 did not include constructs relating to falls-related self-efficacy or balance confidence, 11 assessed other measurement properties, six did not assess measurement properties, two were conducted on different populations, two were abstracts, one was a thesis, one was in citation and four were written in other languages (i.e. Persian, German, Dutch). Thirty-five records were included: 24 full-text articles met eligibility criteria and 11 additional articles from citation tracking, were used to evaluate instrument development (16 studies), content validity (33 studies) and structural validity (14 studies).

### Content validity

#### Quality of instrument development studies

A summary of the studies detailing construct definition, target population, and the intended context of use for the 18 instruments was presented (see Additional file 3). Nine studies were related to scales measuring falls efficacy. Four studies were related to the construct of balance confidence. Three studies were related to scales with the title relating to falls efficacy; however the studies measured concerns about falling rather than constructs relating to falls efficacy or balance confidence.

Concept elicitation was identified as inadequate for 15 instruments because no target population had been involved in their development. For the other instruments (i.e. ABC-16, CONFBal and Mobility Efficacy Scale (MES)), concept elicitation was doubtful because of unclear methods. Among all studies relating to an instrument’s development, only Icon-FES featured cognitive interviews with older adults. However, the quality of cognitive interviews was doubtful because the characteristics of the sample population and methodology of the interview process were not described.

#### Quality and results of content validity studies

Forty-seven studies were reviewed for content validity of the instruments. Thirty-four studies had involved a target population (see Additional file 4), with 13 studies involving professionals (see Additional file 5). There were no studies on the content validity of Gait efficacy scale (GES)-8 found. Among all instruments, ABC-16 had the highest number of 18 studies conducted that involved older adults (32%) and professionals (54%) respectively. For scales involving the target population in assessing content validity, only one study (MFES-13) was of adequate quality to assess its relevance, comprehensibility and comprehensiveness. Two studies on relevance (FES-10 and ABC-6) were of inadequate quality, and one study on comprehensibility (FES-10) was of inadequate quality. Fifteen content validity studies involving target populations were cross-cultural adaptations that included a pretest of the translated instruments. In these studies, 6 studies assessing relevance were of doubtful quality, while 6 studies assessing comprehensibility were also of doubtful quality. All other studies were of either inadequate or indeterminate quality. None of the studies assessed comprehensiveness adequately. A significant number of content validity studies involving patients (44%) were cross-cultural adaptations that included a pre-test of the translated instruments (FES-10, MFES-13, MFES-14, ABC-6, ABC-16) with the largest number of studies on ABC-16 (60%). These studies were of doubtful (47%), inadequate (13%) or indeterminate (40%) quality.

Out of the 13 content validity studies involving professionals, 10 were cross-cultural adaptation studies. Two studies on the original instruments explored the relevance of the FES-10 and the comprehensiveness of the Icon-FES. However, both were of doubtful quality [7, 13]. All studies that had included cross-cultural adaptation research involving 6 instruments (FES-10, MFES-13, MFES-14, ABC-15, ABC-16, Icon-FES), were of doubtful or indeterminate quality.

#### Evidence synthesis for falls efficacy scales

Among all instruments evaluating falls efficacy, MFES-13 had high quality evidence demonstrating sufficient results for relevance (based on one adequate quality study and reviewers’ rating) [31], and insufficient results for comprehensiveness (based on one adequate quality study and reviewers’ rating) [31]. Moderate quality evidence was only available for FES-10, which had sufficient results for relevance (based on one doubtful quality study); MFES-13, which had inconsistent results for comprehensibility (based on one adequate quality study and one doubtful quality study); and MFES-14, which had sufficient results for comprehensibility (based on two doubtful quality studies) [31–34]. For all other related instruments measuring falls efficacy, evidence quality had been generally low to very low (see Additional file 7). There had been no relevant studies of content validity studies and related studies were of inadequate quality based on reviewers’ ratings.

#### Evidence synthesis for balance confidence scales

Among all instruments evaluating balance confidence, moderate quality evidence was only available for the ABC-15. It displayed sufficient results for relevance (based on one content validity study of doubtful quality) [23]. However, insufficient results for comprehensiveness and sufficient results for comprehensibility were supported by very low quality evidence. Similarly, for instruments measuring balance confidence, evidence quality had been generally low to very low (see Additional file 7). There had been no relevant studies of content validity studies and based on reviewers’ ratings, even related studies had shown inadequate quality.

#### Evidence synthesis for scales with titles relating to falls efficacy

Three scales with titles relating to falls efficacy, Icon-FES, FES-I and MES were developed to measure fear of falling and/or concerns about falling [12, 13, 35]. The Icon-FES was the only scale to have been underpinned by moderate-quality evidence to display sufficient results for relevance and comprehensiveness (based on one doubtful quality study) [13]. Other assessments for Icon-FES, FES-I and MES were rated as low to very low by reviewers given the absence of quality within any relevant studies of content validity.

### Structural validity

#### Quality and results of studies

A total of 14 studies (see Additional file 6) assessed structural validity of falls-related self-efficacy (4 studies) [4, 9, 34], balance confidence (8 studies) [23, 36–41] and falls efficacy related titled scales (2 studies) [12, 13]. The majority of authors used exploratory factor analysis (EFA, 72%) [4, 9, 12, 34, 36, 37, 40–42]. The other studies used IRT Rasch model (7%) [38], IRT polytomous model (7%) [23] or more that a single method of analysis (14%) [13, 39]. 93% of the studies were of at least adequate quality, 64% were of high quality and 29% were of adequate quality. Only one study was of inadequate quality, because an insufficient sample size had been used for analysis [37].

#### Evidence synthesis

All studies on FES-10, MFES-14, ABC-6, ABC-15, ABC-16, Icon-FES, FES-I and PAPMFR reported positive results, and provided high-quality evidence of sufficient unidimensionality. All the other instruments displayed indeterminate ratings.

## Discussion

### Development and content validity of falls efficacy related scales

Our synthesised findings from the published literature showed a lack of high quality evidence for falls efficacy-related scales. Of 11 scales specifically measuring falls efficacy and its relevance, only the MFES-13 demonstrated high-quality evidence. However, MFES-13 showed insufficient comprehensiveness and inconsistent results of comprehensibility supported by high and moderate-quality evidence, respectively. The FES-10 and MFES-14 were supported by moderate-quality evidence for both sufficient relevance and comprehensibility. By contrast, both scales had very low-quality evidence supporting their comprehensiveness.

For scales measuring balance confidence, only the ABC-15 had sufficient relevance supported by moderate-quality evidence, with very low-quality evidence supporting both its insufficient comprehensiveness, as well as sufficient comprehensibility. Furthermore, evidential quality for the content validity of the remaining 14 instruments was low to very low. As such, this review demonstrated that current evidence is inadequate in the recommendation of any existing instruments for the measurement of measure balance confidence.

Furthermore, none of the 15 scales designed to assess either balance confidence or falls efficacy offered sufficient quality or consistency of evidence for content validity to support their unreserved use in community-dwelling older adults. Despite their routine contemporary use, only four scales (MFES-13, FES-10, MFES-14 and ABC-15) had been underpinned by partial relevant evidence.

Instruments with titles relating to falls efficacy but measuring other constructs such as fear of falling (FES-I, Icon-FES and MES), had been categorised separately. The FES-I’s developers stated that their instrument assessed concerns about falling, even though the term ‘Falls Efficacy’ had been retained in the title to acknowledge the historical development of the scale [12]. Icon-FES [43], developed from literature on the measures of fear of falling, showed sufficient relevance and comprehensiveness but with only moderate-quality evidential support. Further concurrent research amongst scales of fear of falling would reconcile selection preferences.

### Structural validity

Eight instruments (FES-10, MFES-14, ABC-6, ABC-15, ABC-16, Icon-FES, FES-I and PAPMFR) demonstrated sufficient unidimensionality relating to either falls efficacy or balance confidence, with support from high-quality evidence.

Nevertheless, unidimensionality might not ascertain the construct of interest would be measured adequately, or that no important concepts would be missed, of which had been a fundamental concern, emphasising the pivotal role of content validity within psychometric analyses [30]. Failures in adopting proper methodologies within instrument development, including during concept elicitation or compromised cognitive interviewing in a target population, may lead to confusion in selecting instruments.

**Fig. 2:**
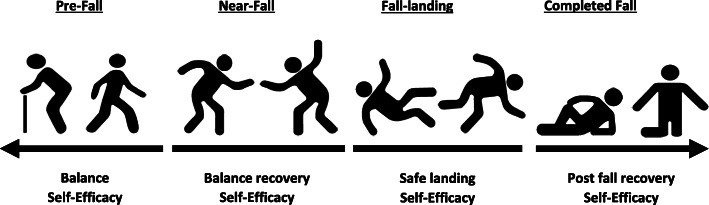
Falls-related Self-Efficacy Continuum Model

Our evaluation of the instruments’ content has identified that the conceptual framework of the constructs of falls efficacy and balance confidence differed amongst instruments and should not be interpreted uniformly. The 11 instruments measuring falls efficacy revealed content containing four domains of self-efficacy which addressed the potential of falling. The four domains may be expressed in a continuum of situational-specific phases of pre-fall, near-fall, fall-landing and a completed fall (Fig. 2). Balance efficacy (or balance confidence) and balance recovery in pre-fall and near-fall phases, respectively, are defined as the perceived abilities to undertake activities of daily living without losing balance and to execute balance recovery manoeuvres so as to prevent falling. Similarly, efficacy in fall-landing, post-fall and completed fall phases, reflect abilities to fall safely, to get (helped) up and to accomplish actions after falling, respectively. This knowledge, acquired through appropriate self-reported instruments, would help researchers and clinicians work with community-dwelling older adults in reconciling their perceived abilities, and to have their actual abilities assessed and trained, through outcome-based emerging rehabilitation work, i.e. perturbation-based balance training and safe falling techniques training programs [44, 45]. While there may not be an all-purpose measure of perceived self-efficacy in managing a range of circumstances surrounding falling adequately, different measures might facilitate greater understanding of the abilities of community-dwelling older adults in managing both falling and personal efficacy effectively.

### Limitations of the study

This review limited its scope to exclude instruments with titles relating to and measuring constructs such as ‘fear’, ‘anxiety’ and ‘activity avoidance’. We were persuaded of the latter constructs’ distinctiveness compared to the review’s focus, and could have had an unrealistic expectation that high-quality evidence about falls efficacy and balance confidence could be derived from them. Furthermore, a language limitation amongst the review team hindered its ability to translate, review and accurately rate the quality of evidence of four articles on ABC-16, written in German, Dutch, and Persian. Similarly, rating of evidence qualities amongst the review articles may have had been hampered inadvertently by the review team not having contacted the respective study authors in seeking clarification about their published descriptions of study designs (e.g. interview methodologies).

## Conclusion

This systematic review had applied the COSMIN methodology to thoroughly assess the content and structural validity of a set of falls efficacy related instruments in community-dwelling older adults. This review highlighted the importance of future research on the development and measurement properties of instruments measuring falls efficacy. Cognitive interviews involving target populations such as community-dwelling older adults, as well as concomitant research into content validation amongst target populations and professionals from all relevant disciplines would be needed to strengthen the evidence for recommending appropriate instruments to measure the intended construct.

## Supplementary Information

**Additional file 1.** Search Strategy. A table detailing the search terms used in the five databases.

**Additional file 2.** Criteria guide to rate studies on structural validity. A criteria guide published by Prinsen and colleagues (2018) which was used to rate studies on structural validity.

**Additional file 3.** Characteristics and quality assessment of the studies on the development of the included instruments. A table detailing information about the included instruments and the quality rating of the concept elicitation done for instrument development.

**Additional file 4.** Characteristics, quality assessment and results of the content validity studies. A table detailing information about content validity studies involving target population.

**Additional file 5.** Characteristics, quality assessment and results of the content validity studies involving professionals. A table detailing information about the content validity studies involving professionals.

**Additional file 6.** Characteristics, quality assessment, and results of the structural validity studies of instruments measuring falls-related self-efficacy or balance confidence in community-dwelling older adults. A table detailing information about the structural validity studies of instruments measuring falls-related self-efficacy or balance confidence in community-dwelling older adults.

**Additional file 7.** Evidence synthesis on the content and structural validity of instruments measuring falls-related self-efficacy or balance confidence in community-dwelling older adults. A table detailing the synthesis of evidence on the content validity and structural validity of falls-related self-efficacy instruments or balance confidence instruments for the community-dwelling older adults.

**Additional file 8.** PRISMA 2009 Checklist. A completed PRISMA checklist.

## Data Availability

All data generated or analysed during this study are included in this published article [and its supplementary information files].

## Abbreviations

FES: Falls Efficacy Scale
MFES: Modified Falls Efficacy Scale
ABC: Activities-specific Balance Confidence Scale
CONFBal: CONFBal scale of balance confidence
FES-I: Falls Efficacy Scale-International
Icon-FES: Iconographical Falls Efficacy Scale
SAFE: The Survey of Activities and Fear of Falling in the Elderly
UICFFM: The University of Illinois at Chicago Fear of Falling Measure
GFFM: Geriatric Fear of Falling Measure
PAPMFR: Perceived Ability to Prevent and Manage Fall Risk
COSMIN: Consensus-based Standards for the Selection of Health Measurement Instruments
PRISMA: Preferred Reporting Items of Systematic Reviews and Meta-Analyses Protocol
GES: Gait Efficacy Scale
EFA: Exploratory Factor Analysis
IRT: Item Response Theory
MES: Mobility Efficacy Scale
