# Supplementary material for: Falls efficacy instruments for community-dwelling older adults: a COSMIN-based systematic review
Source: BMC Geriatr. 2021 Jan 7;21:21. doi: 10.1186/s12877-020-01960-7 (PMC7792090; doi:10.1186/s12877-020-01960-7)
Supplement: Supplementary file 1 — Additional file 1. Search Strategy. A table detailing the search terms used in the five databases. [file 12877_2020_1960_MOESM1_ESM.docx]

**Additional file 1: Search Strategy**

| Cinahl Plus with Full Text via EBSCOhost | |
| --- | --- |
| January 1990 – May 2019 | |
| Results: 655 | |
| Searches | Search Terms |
| 1 | TI-AB=elder* OR TI-AB=senior OR TI-AB=older OR TI-AB= aged |
| 2 | TI-AB=falls efficacy OR TI-AB=falls self-efficacy OR TI-AB=balance confidence OR TI-AB=balance efficacy OR TI-AB=balance self-efficacy OR TI-AB=balance recovery confidence OR TI-AB=balance recovery efficacy OR TI-AB=balance recovery self-efficacy OR (TI-AB=falls AND TI-AB=perceived control OR TI-AB=self-perceived control OR TI-AB=perceived ability OR TI-AB=self-perceived ability) |
| 3 | psychometr* OR observer variation OR reproducib* OR reliab* OR unreliab* OR valid* OR coefficient OR homogeneity OR homogeneous OR “internal consistency” OR psychometr* OR observer variation OR reproducib* OR reliab* OR unreliab* OR valid* OR coefficient OR homogeneity OR homogeneous OR “internal consistency” OR (cronbach* OR cronbach* AND (alpha OR alpha OR Talphas OR alphas)) OR (item OR item AND (correlation* OR correlation* OR selection* OR selection* OR reduction* OR reduction*)) OR agreement OR precision OR imprecision OR “precise values” OR test-retest OR agreement OR precision OR imprecision OR “precise values” OR test-retest OR (test OR test AND retest OR retest) OR (reliab* OR reliab* AND (test OR test OR retest or retest)) OR stability OR interrater OR interrater OR intrarater OR intra-rater OR intertester OR inter-tester OR intratester OR intra-tester OR interobserver OR inter-observer OR intraobserver OR intra-observer OR intertechnician OR inter-technician OR intratechnician OR intra-technician OR interexaminer OR inter-examiner OR intraexaminer OR intra-examiner OR interassay OR inter-assay OR intraassay OR intra-assay OR interindividual OR inter-individual OR intraindividual OR intra-individual OR interparticipant OR inter-participant OR intraparticipant OR intra-participant OR kappa OR kappa’s OR kappas OR repeatab* OR stability OR interrater OR inter-rater OR intrarater OR intra-rater OR intertester OR inter-tester OR intratester OR intra-tester OR interobserver OR inter-observer OR intraobserver OR intra-observer OR intertechnician OR inter-technician OR intratechnician OR intra-technician OR interexaminer OR inter-examiner OR intraexaminer OR intra examiner OR interassay OR inter-assay OR intraassay OR intra-assay OR interindividual OR inter-individual OR intraindividual OR intra-individual OR interparticipant OR inter-participant OR intraparticipant OR intra-participant OR kappa OR kappa’s OR kappas OR repeatab* OR ((replicab* OR replicab* OR repeated OR repeated) AND (measure OR measure OR measures OR measures OR findings OR findings OR result OR result OR results OR results OR test OR test OR tests OR tests)) OR generaliza* OR generalisa* OR concordance OR generaliza* OR generalisa* OR concordance OR (intraclass OR intraclass AND correlation* or correlation*) OR discriminative OR “known group” OR factor analysis OR factor analyses OR dimension* OR subscale* OR discriminative OR “known group” OR factor analysis OR factor analyses OR dimension* OR subscale* OR (multitrait OR multitrait AND scaling OR scaling AND (analysis OR analysis OR analyses OR analyses)) OR item discriminant OR interscale correlation* OR error OR errors OR “individual variability” OR item discriminant OR interscale correlation* OR error OR errors OR “individual variability” OR (variability OR variability AND (analysis OR analysis OR values OR values)) OR (uncertainty OR uncertainty AND (measurement OR measurement OR measuring OR measuring)) OR “standard error of measurement” OR sensitiv* OR responsive* OR “standard error of measurement” OR sensitiv* OR responsive* OR ((minimal OR minimally OR clinical OR clinically OR minimal OR minimally OR clinical OR clinically) AND (important OR significant OR detectable OR important OR significant OR detectable) AND (change OR change OR difference OR difference)) OR (small* OR small* AND (real OR real OR detectable OR detectable) AND (change OR change OR difference OR difference)) OR meaningful change OR “ceiling effect” OR “floor effect” OR “Item response model” OR IRT OR Rasch OR “Differential item functioning” OR DIF OR “computer adaptive testing” OR “item bank” OR “cross-cultural equivalence” OR outcome assessment OR meaningful change OR “ceiling effect” OR “floor effect” OR “Item response model” OR IRT OR Rasch OR “Differential item functioning” OR DIF OR “computer adaptive testing” OR “item bank” OR “cross-cultural equivalence” OR outcome assessment) |
| 4 | AND/#1-#3 |

| MEDLINE via Ebscohost | |
| --- | --- |
| January 1990 – May 2019 | |
| Results: 742 | |
| Searches | Search Terms |
| 1 | TI-AB=elder* OR TI-AB=senior OR TI-AB=older OR TI-AB= aged |
| 2 | TI-AB=falls efficacy OR TI-AB=falls self-efficacy OR TI-AB=balance confidence OR TI-AB=balance efficacy OR TI-AB=balance self-efficacy OR TI-AB=balance recovery confidence OR TI-AB=balance recovery efficacy OR TI-AB=balance recovery self-efficacy OR (TI-AB=falls AND TI-AB=perceived control OR TI-AB=self-perceived control OR TI-AB=perceived ability OR TI-AB=self-perceived ability) |
| 3 | psychometr* OR observer variation OR reproducib* OR reliab* OR unreliab* OR valid* OR coefficient OR homogeneity OR homogeneous OR “internal consistency” OR psychometr* OR observer variation OR reproducib* OR reliab* OR unreliab* OR valid* OR coefficient OR homogeneity OR homogeneous OR “internal consistency” OR (cronbach* OR cronbach* AND (alpha OR alpha OR Talphas OR alphas)) OR (item OR item AND (correlation* OR correlation* OR selection* OR selection* OR reduction* OR reduction*)) OR agreement OR precision OR imprecision OR “precise values” OR test-retest OR agreement OR precision OR imprecision OR “precise values” OR test-retest OR (test OR test AND retest OR retest) OR (reliab* OR reliab* AND (test OR test OR retest or retest)) OR stability OR interrater OR interrater OR intrarater OR intra-rater OR intertester OR inter-tester OR intratester OR intra-tester OR interobserver OR inter-observer OR intraobserver OR intra-observer OR intertechnician OR inter-technician OR intratechnician OR intra-technician OR interexaminer OR inter-examiner OR intraexaminer OR intra-examiner OR interassay OR inter-assay OR intraassay OR intra-assay OR interindividual OR inter-individual OR intraindividual OR intra-individual OR interparticipant OR inter-participant OR intraparticipant OR intra-participant OR kappa OR kappa’s OR kappas OR repeatab* OR stability OR interrater OR inter-rater OR intrarater OR intra-rater OR intertester OR inter-tester OR intratester OR intra-tester OR interobserver OR inter-observer OR intraobserver OR intra-observer OR intertechnician OR inter-technician OR intratechnician OR intra-technician OR interexaminer OR inter-examiner OR intraexaminer OR intra examiner OR interassay OR inter-assay OR intraassay OR intra-assay OR interindividual OR inter-individual OR intraindividual OR intra-individual OR interparticipant OR inter-participant OR intraparticipant OR intra-participant OR kappa OR kappa’s OR kappas OR repeatab* OR ((replicab* OR replicab* OR repeated OR repeated) AND (measure OR measure OR measures OR measures OR findings OR findings OR result OR result OR results OR results OR test OR test OR tests OR tests)) OR generaliza* OR generalisa* OR concordance OR generaliza* OR generalisa* OR concordance OR (intraclass OR intraclass AND correlation* or correlation*) OR discriminative OR “known group” OR factor analysis OR factor analyses OR dimension* OR subscale* OR discriminative OR “known group” OR factor analysis OR factor analyses OR dimension* OR subscale* OR (multitrait OR multitrait AND scaling OR scaling AND (analysis OR analysis OR analyses OR analyses)) OR item discriminant OR interscale correlation* OR error OR errors OR “individual variability” OR item discriminant OR interscale correlation* OR error OR errors OR “individual variability” OR (variability OR variability AND (analysis OR analysis OR values OR values)) OR (uncertainty OR uncertainty AND (measurement OR measurement OR measuring OR measuring)) OR “standard error of measurement” OR sensitiv* OR responsive* OR “standard error of measurement” OR sensitiv* OR responsive* OR ((minimal OR minimally OR clinical OR clinically OR minimal OR minimally OR clinical OR clinically) AND (important OR significant OR detectable OR important OR significant OR detectable) AND (change OR change OR difference OR difference)) OR (small* OR small* AND (real OR real OR detectable OR detectable) AND (change OR change OR difference OR difference)) OR meaningful change OR “ceiling effect” OR “floor effect” OR “Item response model” OR IRT OR Rasch OR “Differential item functioning” OR DIF OR “computer adaptive testing” OR “item bank” OR “cross-cultural equivalence” OR outcome assessment OR meaningful change OR “ceiling effect” OR “floor effect” OR “Item response model” OR IRT OR Rasch OR “Differential item functioning” OR DIF OR “computer adaptive testing” OR “item bank” OR “cross-cultural equivalence” OR outcome assessment) |
| 4 | AND/#1-#3 |

| PsychINFO via Ebscohost | |
| --- | --- |
| January 1990 – May 2019 | |
| Results: 323 | |
| Searches | Search Terms |
| 1 | TI-AB=elder* OR TI-AB=senior OR TI-AB=older OR TI-AB= aged |
| 2 | TI-AB=falls efficacy OR TI-AB=falls self-efficacy OR TI-AB=balance confidence OR TI-AB=balance efficacy OR TI-AB=balance self-efficacy OR TI-AB=balance recovery confidence OR TI-AB=balance recovery efficacy OR TI-AB=balance recovery self-efficacy OR (TI-AB=falls AND TI-AB=perceived control OR TI-AB=self-perceived control OR TI-AB=perceived ability OR TI-AB=self-perceived ability) |
| 3 | psychometr* OR observer variation OR reproducib* OR reliab* OR unreliab* OR valid* OR coefficient OR homogeneity OR homogeneous OR “internal consistency” OR psychometr* OR observer variation OR reproducib* OR reliab* OR unreliab* OR valid* OR coefficient OR homogeneity OR homogeneous OR “internal consistency” OR (cronbach* OR cronbach* AND (alpha OR alpha OR Talphas OR alphas)) OR (item OR item AND (correlation* OR correlation* OR selection* OR selection* OR reduction* OR reduction*)) OR agreement OR precision OR imprecision OR “precise values” OR test-retest OR agreement OR precision OR imprecision OR “precise values” OR test-retest OR (test OR test AND retest OR retest) OR (reliab* OR reliab* AND (test OR test OR retest or retest)) OR stability OR interrater OR interrater OR intrarater OR intra-rater OR intertester OR inter-tester OR intratester OR intra-tester OR interobserver OR inter-observer OR intraobserver OR intra-observer OR intertechnician OR inter-technician OR intratechnician OR intra-technician OR interexaminer OR inter-examiner OR intraexaminer OR intra-examiner OR interassay OR inter-assay OR intraassay OR intra-assay OR interindividual OR inter-individual OR intraindividual OR intra-individual OR interparticipant OR inter-participant OR intraparticipant OR intra-participant OR kappa OR kappa’s OR kappas OR repeatab* OR stability OR interrater OR inter-rater OR intrarater OR intra-rater OR intertester OR inter-tester OR intratester OR intra-tester OR interobserver OR inter-observer OR intraobserver OR intra-observer OR intertechnician OR inter-technician OR intratechnician OR intra-technician OR interexaminer OR inter-examiner OR intraexaminer OR intra examiner OR interassay OR inter-assay OR intraassay OR intra-assay OR interindividual OR inter-individual OR intraindividual OR intra-individual OR interparticipant OR inter-participant OR intraparticipant OR intra-participant OR kappa OR kappa’s OR kappas OR repeatab* OR ((replicab* OR replicab* OR repeated OR repeated) AND (measure OR measure OR measures OR measures OR findings OR findings OR result OR result OR results OR results OR test OR test OR tests OR tests)) OR generaliza* OR generalisa* OR concordance OR generaliza* OR generalisa* OR concordance OR (intraclass OR intraclass AND correlation* or correlation*) OR discriminative OR “known group” OR factor analysis OR factor analyses OR dimension* OR subscale* OR discriminative OR “known group” OR factor analysis OR factor analyses OR dimension* OR subscale* OR (multitrait OR multitrait AND scaling OR scaling AND (analysis OR analysis OR analyses OR analyses)) OR item discriminant OR interscale correlation* OR error OR errors OR “individual variability” OR item discriminant OR interscale correlation* OR error OR errors OR “individual variability” OR (variability OR variability AND (analysis OR analysis OR values OR values)) OR (uncertainty OR uncertainty AND (measurement OR measurement OR measuring OR measuring)) OR “standard error of measurement” OR sensitiv* OR responsive* OR “standard error of measurement” OR sensitiv* OR responsive* OR ((minimal OR minimally OR clinical OR clinically OR minimal OR minimally OR clinical OR clinically) AND (important OR significant OR detectable OR important OR significant OR detectable) AND (change OR change OR difference OR difference)) OR (small* OR small* AND (real OR real OR detectable OR detectable) AND (change OR change OR difference OR difference)) OR meaningful change OR “ceiling effect” OR “floor effect” OR “Item response model” OR IRT OR Rasch OR “Differential item functioning” OR DIF OR “computer adaptive testing” OR “item bank” OR “cross-cultural equivalence” OR outcome assessment OR meaningful change OR “ceiling effect” OR “floor effect” OR “Item response model” OR IRT OR Rasch OR “Differential item functioning” OR DIF OR “computer adaptive testing” OR “item bank” OR “cross-cultural equivalence” OR outcome assessment) |
| 4 | AND/#1-#3 |

| SCOPUS | |
| --- | --- |
| January 1990 – May 2019 | |
| Results: 135 | |
| Searches | Search Terms |
| 1 | ( ( TITLE ( elder* ) AND PUBYEAR > 1989 ) OR ( TITLE ( senior ) AND PUBYEAR > 1989 ) OR ( TITLE ( older ) AND PUBYEAR > 1989 ) OR ( TITLE ( aged ) AND PUBYEAR > 1989 ) ) |
| 2 | TITLE ( falls AND efficacy ) AND PUBYEAR > 1989 ) OR ( TITLE ( falls AND self-efficacy ) AND PUBYEAR > 1989 ) OR ( TITLE ( balance AND confidence ) AND PUBYEAR > 1989 ) OR ( TITLE ( balance AND efficacy ) AND PUBYEAR > 1989 ) OR ( TITLE ( balance AND self-efficacy ) AND PUBYEAR > 1989 ) OR ( TITLE ( balance AND recovery AND confidence ) AND PUBYEAR > 1989 ) OR ( TITLE ( balance AND recovery AND efficacy ) AND PUBYEAR > 1989 ) OR ( TITLE ( balance AND recovery AND self-efficacy ) AND PUBYEAR > 1989 ) ) OR ( ( TITLE-ABS ( falls ) AND PUBYEAR > 1989 ) AND ( ( TITLE-ABS ( perceived AND control ) AND PUBYEAR > 1989 ) OR ( TITLE-ABS ( self-perceived AND control ) AND PUBYEAR > 1989 ) OR ( TITLE-ABS ( perceived AND ability ) AND PUBYEAR > 1989 ) OR ( TITLE-ABS ( self-perceived AND ability ) AND PUBYEAR > 1989 ) ) ) ) |
| 3 | ( TITLE ( psychometr* ) OR TITLE ( observer AND variation ) OR TITLE ( reproducib* ) OR TITLE ( reliab* ) OR TITLE ( unreliab* ) OR TITLE ( valid* ) OR TITLE ( coefficient ) OR TITLE ( homogeneity ) OR TITLE ( homogeneous ) OR TITLE ( "internal consistency" ) OR ABS ( psychometr* ) OR ABS ( observer AND variation ) OR ABS ( reproducib* ) OR ABS ( reliab* ) OR ABS ( unreliab* ) OR ABS ( valid* ) OR ABS ( coefficient ) OR ABS ( homogeneity ) OR ABS ( homogeneous ) OR ABS ( "internal consistency" ) OR ( TITLE ( cronbach* ) OR TITLE ( cronbach* ) AND ( TITLE ( alpha ) OR ABS ( alpha ) OR TITLE ( alphas ) OR ABS ( alphas ) ) ) OR ( TITLE ( item ) OR ABS ( item ) AND ( TITLE ( correlation* ) OR ABS ( correlation* ) OR TITLE ( selection* ) OR ABS ( selection* ) OR TITLE ( reduction* ) OR ABS ( reduction* ) ) ) OR TITLE ( agreement ) OR TITLE ( precision ) OR TITLE ( imprecision ) OR TITLE ( "precise values" ) OR TITLE ( test-retest ) OR ABS ( agreement ) OR ABS ( precision ) OR ABS ( imprecision ) OR ABS ( "precise values" ) OR ABS ( test-retest ) OR ( TITLE ( test ) OR ABS ( test ) AND TITLE ( retest ) OR ABS ( retest ) ) OR ( TITLE ( reliab* ) OR ( ABS ( reliab* ) AND TITLE ( test ) OR ABS ( test ) OR TITLE ( retest ) OR ABS ( retest ) ) ) OR TITLE ( stability ) OR TITLE ( interrater ) OR TITLE ( interrater ) OR TITLE ( intrarater ) OR TITLE ( intra-rater ) OR TITLE ( intertester ) OR TITLE ( inter-tester ) OR TITLE ( intratester ) OR TITLE ( intra-tester ) OR TITLE ( interobserver ) OR TITLE ( inter-observer ) OR TITLE ( intraobserver ) OR TITLE ( intra-observer ) OR TITLE ( intertechnician ) OR TITLE ( inter-technician ) OR TITLE ( intratechnician ) OR TITLE ( intra-technician ) OR TITLE ( interexaminer ) OR TITLE ( inter-examiner ) OR TITLE ( intraexaminer ) OR TITLE ( intra-examiner ) OR TITLE ( interassay ) OR TITLE ( inter-assay ) OR TITLE ( intraassay ) OR TITLE ( intra-assay ) OR TITLE ( interindividual ) OR TITLE ( inter-individual ) OR TITLE ( intraindividual ) OR TITLE ( intra-individual ) OR TITLE ( interparticipant ) OR TITLE ( inter-participant ) OR TITLE ( intraparticipant ) OR TITLE ( intra-participant ) OR TITLE ( kappa ) OR TITLE ( kappa's ) OR TITLE ( kappas ) OR TITLE ( repeatab* ) OR ABS ( stability ) OR ABS ( interrater ) OR ABS ( inter-rater ) OR ABS ( intrarater ) OR ABS ( intra-rater ) OR ABS ( intertester ) OR ABS ( inter-tester ) OR ABS ( intratester ) OR ABS ( intra-tester ) OR ABS ( interobserver ) OR ABS ( inter-observer ) OR ABS ( intraobserver ) OR ABS ( intra-observer ) OR ABS ( intertechnician ) OR ABS ( inter-technician ) OR ABS ( intratechnician ) OR ABS ( intra-technician ) OR ABS ( interexaminer ) OR ABS ( inter-examiner ) OR ABS ( intraexaminer ) OR ABS ( intra-examiner ) OR ABS ( interassay ) OR ABS ( inter-assay ) OR ABS ( intraassay ) OR ABS ( intra-assay ) OR ABS ( interindividual ) OR ABS ( inter-individual ) OR ABS ( intraindividual ) OR ABS ( intra-individual ) OR ABS ( interparticipant ) OR ABS ( inter-participant ) OR ABS ( intraparticipant ) OR ABS ( intra-participant ) OR ABS ( kappa ) OR ABS ( kappa's ) OR ABS ( kappas ) OR ABS ( repeatab* ) OR ( ( TITLE ( replicab* ) OR ABS ( replicab* ) OR TITLE ( repeated ) OR ABS ( repeated ) ) AND ( TITLE ( measure ) OR ABS ( measure ) OR TITLE ( measures ) OR ABS ( measures ) OR TITLE ( findings ) OR TITLE ( result ) OR ABS ( result ) OR TITLE ( results ) OR ABS ( results ) OR TITLE ( test ) OR ABS ( test ) OR TITLE ( tests ) OR ABS ( tests ) ) ) OR TITLE ( generaliza* ) OR TITLE ( generalisa* ) OR TITLE ( concordance ) OR ABS ( generaliza* ) OR ABS ( generalisa* ) OR ABS ( concordance ) OR ( TITLE ( intraclass ) OR ABS ( intraclass ) AND TITLE ( correlation* ) OR ABS ( correlation* ) ) OR TITLE ( discriminative ) OR TITLE ( "known group" ) OR TITLE ( factor AND analysis ) OR TITLE ( factor AND analyses ) OR TITLE ( dimension* ) OR TITLE ( subscale* ) OR ABS ( discriminative ) OR ABS ( "known group" ) OR ABS ( factor AND analysis ) OR ABS ( factor AND analyses ) OR ABS ( dimension* ) OR ABS ( subscale* ) OR ( TITLE ( multitrait ) OR ABS ( multitrait ) OR ABS ( scaling ) AND ( TITLE ( analysis ) OR ABS ( analysis ) OR TITLE ( analyses ) OR ABS ( analyses ) ) ) OR TITLE ( item AND discriminant ) OR TITLE ( interscale AND correlation* ) OR TITLE ( error ) OR TITLE ( errors ) OR TITLE ( "individual variability" ) OR ABS ( item AND discriminant ) OR ABS ( interscale AND correlation* ) OR ABS ( error ) OR ABS ( errors ) OR ABS ( "individual variability" ) OR ( TITLE ( variability ) OR ABS ( variability ) AND ( TITLE ( analysis ) OR ABS ( analysis ) OR TITLE ( values ) OR ABS ( values ) ) ) OR ( TITLE ( uncertainty ) OR ABS ( uncertainty ) AND ( TITLE ( measurement ) OR ABS ( measurement ) OR TITLE ( measuring ) OR ABS ( measuring ) ) ) OR TITLE ( "standard error of measurement" ) OR TITLE ( sensitiv* ) OR TITLE ( responsive* ) OR ABS ( "standard error of measurement" ) OR ABS ( sensitiv* ) OR ABS ( responsive* ) OR ( ( TITLE ( minimal ) OR TITLE ( minimally ) OR TITLE ( clinical ) OR TITLE ( clinically ) OR ABS ( minimal ) OR ABS ( minimally ) OR ABS ( clinical ) OR ABS ( clinically ) ) AND ( TITLE ( important ) OR TITLE ( significant ) OR TITLE ( detectable ) OR ABS ( important ) OR ABS ( significant ) OR ABS ( detectable ) ) AND ( TITLE ( change ) OR ABS ( change ) OR TITLE ( difference ) OR ABS ( difference ) ) ) OR ( TITLE ( small* ) OR ABS ( small* ) AND ( TITLE ( real ) OR ABS ( real ) OR TITLE ( detectable ) OR ABS ( detectable ) AND ( TITLE ( change ) OR ABS ( change ) OR TITLE ( difference ) OR ABS ( difference ) ) ) ) OR TITLE ( meaningful AND change ) OR TITLE ( "ceiling effect" ) OR TITLE ( "floor effect" ) OR TITLE ( "Item response model" ) OR TITLE ( irt ) OR TITLE ( rasch ) OR TITLE ( "Differential item functioning" ) OR TITLE ( dif ) OR TITLE ( "computer adaptive testing" ) OR TITLE ( "item bank" ) OR TITLE ( "cross-cultural equivalence" ) OR TITLE ( outcome AND assessment ) OR ABS ( meaningful AND change ) OR ABS ( "ceiling effect" ) OR ABS ( "floor effect" ) OR ABS ( "Item response model" ) OR ABS ( irt ) OR ABS ( rasch ) OR ABS ( "Differential item functioning" ) OR ABS ( dif ) OR ABS ( "computer adaptive testing" ) OR ABS ( "cross-cultural Equivalence" ) OR ABS ( outcome AND assessment ) AND PUBYEAR > 1989 ) |
| 4 | AND/#1-#3 |

| Web of Science Core Collection | |
| --- | --- |
| January 1990 – May 2019 | |
| Results: 203 | |
| Searches | Search Terms |
| 1 | TS=elder* OR TS=senior OR TS=older OR TS=aged |
| 2 | TI=falls efficacy OR TI=falls self-efficacy OR TI=balance confidence OR TI=balance efficacy OR TI=balance self-efficacy OR TI=balance recovery confidence OR TI=balance recovery efficacy OR TI=balance recovery self-efficacy OR (TS=falls AND TI=perceived control OR TI=self-perceived control OR TI=perceived ability OR TI=self-perceived ability) |
| 3 | TI=psychometr* OR TI=observer variation OR TI=reproducib* OR TI= reliab* OR TI=unreliab* OR TI=valid* OR TI=coefficient OR TI=homogeneity OR TI=homogeneous OR TI=“internal consistency” OR AB=psychometr* OR AB=observer variation OR AB=reproducib* OR AB=reliab* OR AB=unreliab* OR AB=valid* OR AB=coefficient OR AB=homogeneity OR AB=homogeneous OR AB=“internal consistency” OR (TI=cronbach* OR AB=cronbach* AND (TI=alpha OR AB=alpha OR TI=alphas OR AB=alphas)) OR (TI=item OR AB=item AND (TI=correlation* OR AB=correlation* OR TI=selection* OR AB=selection* OR TI=reduction* OR AB=reduction*)) OR TI=agreement OR TI=precision OR TI=imprecision OR TI=“precise values” OR TI=test-retest OR AB=agreement OR AB=precision OR AB=imprecision OR AB=“precise values” OR AB=test-retest OR (TI=test OR AB=test AND TI=retest OR AB=retest) OR (TI=reliab* OR AB=reliab* AND (TI=test OR AB=test OR TI=retest or AB=retest)) OR TI=stability OR TI=interrater OR TI=interrater OR TI=intrarater OR TI=intra-rater OR TI=intertester OR TI=inter-tester OR TI=intratester OR TI=intra-tester OR TI=interobserver OR TI=inter-observer OR TI=intraobserver OR TI=intra-observer OR TI=intertechnician OR TI=inter-technician OR TI=intratechnician OR TI=intra-technician OR TI=interexaminer OR TI=inter-examiner OR TI=intraexaminer OR TI=intra-examiner OR TI=interassay OR TI=inter-assay OR TI=intraassay OR TI=intra-assay OR TI=interindividual OR TI=inter-individual OR TI=intraindividual OR TI=intra-individual OR TI=interparticipant OR TI=inter-participant OR TI=intraparticipant OR TI=intra-participant OR TI=kappa OR TI=kappa’s OR TI=kappas OR TI=repeatab* OR AB=stability OR AB=interrater OR AB=inter-rater OR AB=intrarater OR AB=intra-rater OR AB=intertester OR AB=inter-tester OR AB=intratester OR AB=intra-tester OR AB=interobserver OR AB=inter-observer OR AB=intraobserver OR AB=intra-observer OR AB=intertechnician OR AB=inter-technician OR AB=intratechnician OR AB=intra-technician OR AB=interexaminer OR AB=inter-examiner OR AB=intraexaminer OR AB=intra examiner OR AB=interassay OR AB=inter-assay OR AB=intraassay OR AB=intra-assay OR AB=interindividual OR AB=inter-individual OR AB=intraindividual OR AB=intra-individual OR AB=interparticipant OR AB=inter-participant OR AB=intraparticipant OR AB=intra-participant OR AB=kappa OR AB=kappa’s OR AB=kappas OR AB=repeatab* OR ((TI=replicab* OR AB=replicab* OR TI=repeated OR AB=repeated) AND (TI=measure OR AB=measure OR TI=measures OR AB=measures OR TI=findings OR AB=findings OR TI=result OR AB=result OR TI=results OR AB=results OR TI=test OR AB=test OR TI=tests OR AB=tests)) OR TI=generaliza* OR TI=generalisa* OR TI=concordance OR AB=generaliza* OR AB=generalisa* OR AB=concordance OR (TI=intraclass OR AB=intraclass AND TI=correlation* or AB=correlation*) OR TI=discriminative OR TI=“known group” OR TI=factor analysis OR TI=factor analyses OR TI=dimension* OR TI=subscale* OR AB=discriminative OR AB=“known group” OR AB=factor analysis OR AB=factor analyses OR AB=dimension* OR AB=subscale* OR (TI=multitrait OR AB=multitrait AND TI=scaling OR AB=scaling AND (TI=analysis OR AB=analysis OR TI=analyses OR AB=analyses)) OR TI=item discriminant OR TI=interscale correlation* OR TI=error OR TI=errors OR TI=“individual variability” OR AB=item discriminant OR AB=interscale correlation* OR AB=error OR AB=errors OR AB=“individual variability” OR (TI=variability OR AB=variability AND (TI=analysis OR AB=analysis OR TI=values OR AB=values)) OR (TI=uncertainty OR AB=uncertainty AND (TI=measurement OR AB=measurement OR TI=measuring OR AB=measuring)) OR TI=“standard error of measurement” OR TI=sensitiv* OR TI=responsive* OR AB=“standard error of measurement” OR AB=sensitiv* OR AB=responsive* OR ((TI=minimal OR TI=minimally OR TI=clinical OR TI=clinically OR AB=minimal OR AB=minimally OR AB=clinical OR AB=clinically) AND (TI=important OR TI=significant OR TI=detectable OR AB=important OR AB=significant OR AB=detectable) AND (TI=change OR AB=change OR TI=difference OR AB=difference)) OR (TI=small* OR AB=small* AND (TI=real OR AB=real OR TI=detectable OR AB=detectable) AND (TI=change OR AB=change OR TI=difference OR AB=difference)) OR TI=meaningful change OR TI=“ceiling effect” OR TI=“floor effect” OR TI=“Item response model” OR TI=IRT OR TI=Rasch OR TI=“Differential item functioning” OR TI=DIF OR TI=“computer adaptive testing” OR TI=“item bank” OR TI=“cross-cultural equivalence” OR TI=outcome assessment OR AB=meaningful change OR AB=“ceiling effect” OR AB=“floor effect” OR AB=“Item response model” OR AB=IRT OR AB=Rasch OR AB=“Differential item functioning” OR AB=DIF OR AB=“computer adaptive testing” OR AB=“item bank” OR AB=“cross-cultural equivalence” OR AB=outcome assessment |
| 4 | AND/#1-#3 |
