# Supplementary material for: Falls efficacy instruments for community-dwelling older adults: a COSMIN-based systematic review
Source: BMC Geriatr. 2021 Jan 7;21:21. doi: 10.1186/s12877-020-01960-7 (PMC7792090; doi:10.1186/s12877-020-01960-7)
Supplement: Supplementary file 2 — Additional file 2. Criteria guide to rate studies on structural validity. A criteria guide published by Prinsen and colleagues (2018) which was used to rate studies on structural validity. [file 12877_2020_1960_MOESM2_ESM.docx]

**Additional file 2: Criteria guide to rate studies on structural validity**

Criteria used in this systematic review to determine if the results of each study displayed positive, negative or unknown unidimensionality on the instruments. These criteria are an updated consensus-based criteria on structural validity published by Prinsen and colleagues (2018)

| Rating | Criteria |
| --- | --- |
| Satisfactory  + | CTT  CFA: CFI or TLI or comparable measure > 0.95 OR RMSEA < 0.06 OR SRMR < 0.08a  IRT/Rasch  No violation of unidimensionalityb: CFI or TLI or comparable measure > 0.95 OR RMSEA < 0.06  OR SRMR < 0.08  AND  no violation of local independence: residual correlations among the items after controlling for the  dominant factor < 0.20 OR Q3’s < 0.37  AND  no violation of monotonicity: adequate looking graphs OR item scalability > 0.30  AND  adequate model fit  IRT: χ2 > 0.001  Rasch: infit and outfit mean squares ≥ 0.5 and ≤ 1.5 OR Z-standardized values > −2 and < 2 |
| Unsatisfactory  − | Criteria for ‘+’ not met |
| Unknown  ? | CTT: not all information for ‘+’ reported  IRT/Rasch: model fit not reported |

**Footnotes**

CTT = Classical Test Theory; CFA = Confirmatory Factor Analysis; CFI = Comparative fit Index; TLI = Tucker-Lewis index; RMSEA = Root Mean Square Error of Approximation; SRMR = Standardized Root Mean Square Residual; IRT = Item Response Theory
