# Supplementary material for: Falls efficacy instruments for community-dwelling older adults: a COSMIN-based systematic review
Source: BMC Geriatr. 2021 Jan 7;21:21. doi: 10.1186/s12877-020-01960-7 (PMC7792090; doi:10.1186/s12877-020-01960-7)
Supplement: Supplementary file 3 — Additional file 3. Characteristics and quality assessment of the studies on the development of the included instruments. A table detailing information about the included instruments and the quality rating of the concept elicitation done for instrument development. [file 12877_2020_1960_MOESM3_ESM.docx]

**Additional file 3: Characteristics and quality assessment of the studies on the development of the included instruments**

|  |  |  |  |  |  | Concept elicitation study | |
| --- | --- | --- | --- | --- | --- | --- | --- |
| **Name abbreviation** | **Reference** | **Primary language** | **Construct definition** | **Target population** | **Intended context of use** | **COSMIN quality rating** | **Were patients involved?** |
| **List of falls efficacy scales** | | | | | | | |
| FES-10 | Tinetti 1990 | English (US) | Fall-related self-efficacy as the confidence in performing common daily activities without falling. ‘Low perceived self-efficacy or confidence at avoiding fall’ can be operationally defined as ‘fear of falling’ | Community-dwelling older adults | Assess ‘fear of falling’ interpreted from the total score determined from falls-related self-efficacy. Guide to clinical practice and research. | Inadequate^a^ | No |
| MFES-11 | Edwards 2008 | French | Probably like FES | Community-dwelling older adults | Clinical practice and research to assess confidence and fear of falling to perform challenging activities of daily living including the use of stairs in the home and community without falling | Inadequate^a^ | No |
| MFES-12 | Tennstedt 1998 | English (US) | Probably like FES | Community-dwelling older adults | Probably like FES | Inadequate^a^ | No |
| MFES-13 | Hellstrom 1998 | Swedish | Assess confidence in completing activity without falling | Stroke | Clinical practice and research to assess confidence to perform basic and instrumental activities of daily living without falling. | Inadequate^a^ | No |
| MFES-14 | Hill 1996 | English (Australia) | Probably like FES | Community-dwelling older adults with and without balance dysfunction | Simple, quick, easy-to-administer clinical evaluation for older people | Inadequate^a^ | No |
| PAPMFR | Yoshikawa 2019 | English (US) | Perceived ability to prevent and manage falls | Community –dwelling older adults | Status assessment in clinical practice | Inadequate^a^ | No |
| GES-8 | Rosengren 1998 | English (US) | Probably like GES-10 | Community-dwelling older adults | Probably like GES-10 | Inadequate^a^ | No |
| GES-10 | McAuley 1997 | English (US) | Assessing perceived walking ability to negotiate stairs, curbs and object in their path without falling | Community-dwelling older adults | Clinical trials to study confidence to perform in situation involving walking or stepping without falling | Inadequate^a^ | No |
| PCOF | Tennstedt 1998 | English (US) | Assess perceived control over falling | Community-dwelling older adults | Brief assessment to determine attitude and self-efficacy relating to falls | Inadequate^a^ | No |
| PAMF | Tennstedt 1998 | English (US) | Assessed perceived ability to manage risk of falls or actual falls | Community-dwelling older adults | Brief assessment to determine attitude and self-efficacy relating to falls | Inadequate^a^ | No |
| BSPT | Shumway Cook 1997 | English (US) | Assessing confidence in performing basic activities of daily living and instrumental activities without fear of loss of balance | Community-dwelling older adults | Probably like FES-10 | Inadequate^a^ | No |
| **List of balance confidence scales** | | | | | | | |
| ABC-6 | Peretz 2006 | Hebrew | Assessing fear of falling and balance confidence focusing on a narrower spectrum of activity difficulty | Older adults with HLGD and older adults with PD | Clinical evaluation, management and care of patients with movement disorders | Inadequate^a^ | No |
| ABC-15 | Filiatrault 2007 | English (Canada) | Probably like ABC-16 | Community-dwelling older adults | Probably like ABC-16 | Inadequate^a^ | No |
| ABC-16 | Powell 1995 | English (Canada) | Perceived ability in performing ‘situation-specific’ activities of daily living in a wide continuum of activity difficulty without losing balance or becoming unsteady | High functioning community-dwelling older adults | Clinical practice and research involving high functioning older people | Doubtful^b^ | Yes |
| CONFBal | Simpson 2009 | English (UK) | Perceived ability to engage in everyday functional tasks without losing balance | General older adults and patient population with a greater range of health and mobility problems | Clinical rehabilitation practice to determine functional-activity-related balance confidence | Doubtful^b^ | Yes |
| **List of scales not measuring falls efficacy or balance confidence** | | | | | | | |
| Icon-FES | Delbaere 2011 | English (Australia) | Probably like FES-I | Community-dwelling older adults | Use of pictures to provide clear, unambiguous contexts in a broad range of activities. Use as a treatment utility tool as part of cognitive behavioural therapy program. | Inadequate^a^ | No |
| FES-I | Yardley 2005 | English (UK) | Assess fear of falling or “concerns about falling” relating to basic and more demanding activities both physical and social. | Community-dwelling older adults | Clinical trials to study fear of falling | Inadequate^a^ | No |
| MES | Lusardi 1997 | English (US) | Assess concerns about falling when performing a variety of challenging activities | Community-dwelling older adults | Intervention planning and outcome evaluation in rehabilitation of older adults | Doubtful^b^ | Yes |

**Footnotes**

Rating of standards:

Inadequate^a^ – Evidence rated not adequate on the quality aspect of the study because target population were not involved

Doubtful^b^ – Evidence rated doubtful on the quality aspect of the study (not inadequate) because methods were not clearly described in the study

FES: Falls Efficacy Scale. MFES: Modified Falls Efficacy Scale. PAPMFR: Perceived Ability to Prevent and Manage Fall Risks. GES: Gait Efficacy Scale. PCOF: Perceived Control Over Falling. PAMF: Perceived Ability to Manage Risk of Falls or Actual Falls. BSPT: Balance Self-Perception Test. ABC: Activities-specific Balance Confidence. CONFBal: CONFBal scale of balance confidence. Icon-FES: Iconographical Falls Efficacy Scale. FES-I: Falls Efficacy Scale-International. MES: Mobility Efficacy Scale. HLGD: High Level Gait Disorders. PD: Parkinson’s Disease.
