# Supplementary material for: Falls efficacy instruments for community-dwelling older adults: a COSMIN-based systematic review
Source: BMC Geriatr. 2021 Jan 7;21:21. doi: 10.1186/s12877-020-01960-7 (PMC7792090; doi:10.1186/s12877-020-01960-7)
Supplement: Supplementary file 4 — Additional file 4. Characteristics, quality assessment and results of the content validity studies. A table detailing information about content validity studies involving target population. [file 12877_2020_1960_MOESM4_ESM.docx]

**Additional file 4: Characteristics, quality assessment and results of the content validity studies**

| **Name abbreviation** | **Language**  **(Country)** | **N** | **Female (%)** | **Age** | **Other** | **RE** | **CB** | **CV** | **Remarks** |
| --- | --- | --- | --- | --- | --- | --- | --- | --- | --- |
| **List of falls efficacy scales** | | | | | | | | | |
| FES-10 | English (US) | 18 | 78 | 79 ± 12 | One third lived in the community and two third resided in intermediate care facilities | Inadequate | Inadequate | / | Very few subjects expressed low confidence in grooming or toileting which these items were then replaced by light house-keeping and simple shopping.  All participants were able to complete the 10-point scale without difficulty |
|  | English (US) | 1103 | 73 | 79.6 ± 5.2 | Community -living residents. 69% lives alone. 83% needing assistance with daily tasks such as shopping, cleaning, and providing transportation. 39% experienced one or more falls in the previous year. | ? | ? | ? | FES scoring scale was modified with the low score corresponding to low, rather than high, confidence. Tendency of respondents to relate self-efficacy with actual performance of function than fear of falling. |
|  | English (UK) | >200 |  |  | Subjects involved in falls related research projects | ? | ? | ? | The Anglicised version of the FES in which "cabinets or closets" was changed to "cupboards". No problems identified from participants in falls-related research projects |
| MFES-11 | French (Canada) | 551 | 76 | 73.9 ± 7.56 | 57.5% lived alone. 19.9% use a mobility device such as a cane. 32.8% experience a fall in the past year | ? | ? | ? | No results reported |
| MFES-12 | English (US) | 434 | 89.6 | 77.8 ± 7.71 | 24.9% had one or more falls in the previous three months | ? | ? | ? | No results reported |
| MFES-13 | Swedish (Sweden) | 30 | 56.6 | 81 ± 5.5 | 36.7% lived alone. 80% use a walking aid  40% had a previous stroke; 40% had previous hip fracture; 20% had medical issues such as lower leg amputation, cancer, diverticulitis or myocardial infarction | Adequate | Adequate | Adequate | The ambiguous questions gave participants a great scope of interpretations.  All participants were able to complete the scale. Most items were relevant. Some items may be less relevant for active high functioning elderly person. The scale is less suitable for active elderly.  More than half suggested activities of higher degree of difficulty, recognising the activities were too basic. |
|  | Persian (Iran) | 10 |  |  |  | Doubtful | Doubtful | / | No difficulty in perception of the items and general instructions. |
|  | Swedish (Sweden) | 30 | 33.3 | 65 ± 11 | Sustained a stroke 4-81 months. 66.7% need manual assistance or walking aid | ? | ? | ? | No results reported |
| MFES-14 | Serbian (Serbia) | 10 | 100 | >65 | Community-dwelling women with osteoporosis | Doubtful | Doubtful | / | Some minor changes were made with no further details provided |
|  | French (France) | 25 | 63.2 | 73.5 ± 8.1 |  | Doubtful | Doubtful | / | No results reported |
|  | English (Australia) | 179 | 79.9 | 79.2 ± 6.6 (n=68 with falls and balance issues)  74 ± 4.1 (n=111 healthy older adults) | 46.4% lived alone. 38% experienced one fall or more in the last twelve months. | ? | ? | ? | No results reported |
| PAPMFR | English (US) | 522 | 82.4 | 76.45 ± 7.79 | 51.3% lived alone. 18% had at least one fall in the past month. | ? | ? | ? | No results reported |
| GES-10 | English (US) | 58 | 77.6 | 70.97 ± 6.25 |  | ? | ? | ? | Gait-efficacy measure and the FES showed substantial overlap with each other |
| PCOF | English (US) | 434 | 89.6 | 77.8 ± 7.71 | 24.9% had one or more falls in the previous three months | ? | ? | ? | No results reported |
| PAMF | English (US) | 434 | 89.6 | 77.8 ± 7.71 | 24.9% had one or more falls in the previous three months | ? | ? | ? | No results reported |
| BSPT | English (US) | 105 | 73.3 | 78 ± 8 (n=21 fallers)  80 ± 8 (n=32 partial adherent exercise group)  79 ± 8 (n=52 fully adherent exercise group) | 9% needing an assistive device (n=21)  56% needing an assistive device (n=32)  46% needing an assistive device (n=52) | ? | ? | ? | No results reported |
| **List of balance confidence scales** | | | | | | | | | |
| ABC-6 | Hebrew (Tel Aviv) | 157 | Elderly with HLGD (74.3%)  Elderly with PD (36.8%)  Healthy elderly (54.4%) | Elderly with HLGD (78±5)  Elderly with PD (72±6)  Healthy elderly (75±6) | 44.6% (70) has HLGD,  12.1% (19) has PD  43.3% (68) are elderly free of any clinical impairment likely to affect their gait  All were mobile, independent and did not have dementia, a history of stroke or any acute disease. | Inadequate | / | / | 6 items were selected from the group with HLGD and PD scoring the lowest in ABC-16. The items selected assume to reflect a higher fear of falling.  The shortened version focuses on a narrower spectrum of activity difficulty, emphasizing situations that are most challenging to postural control. Takes approximately 50% of the time required to complete the ABC-16 Hebrew version |
|  | English (US) | 35 |  | 72.86 ± 1.05 | 45.7% community-dwelling older adults reported one or more falls within the last 12 months. | ? | ? | ? | Items can be used to measure balance confidence in healthy community-dwelling older adults activities for a narrow spectrum of activities |
| ABC-15 | English (Canada) | 197 | 84 | 73.9 ± 7.4 | 38% experience 1 or more falls in the previous 12 months | ? | ? | ? | No results reported |
| ABC-16 | Chinese-Mandarin (China) | 61 | 57 | 76.3 ± 7.5 | All were able to stand bipedally for 30 seconds or more.  16% needed aids for outdoor activities,  21% had a history of falling within the past six months. | ? | ? | ? | No results reported |
|  | Icelandic (Iceland) | 5 |  |  | 3 urban and 2 rural volunteers | Doubtful | Doubtful | / | No need for further modification |
|  | Brazilian-Portugese (Brazil) | 20 |  |  | Healthy elderly adults | / | Doubtful | / | None of the respondents reported doubts about the content |
|  | English (Canada) | 602 | 74 |  |  | ? | ? | ? | Items of ABC are relevant for the assessment of balance confidence with most noninstitutionalised older adults. |
|  | English (Canada) | 60 | 72 | 65 - 95 | 53% lived alone; 37% lived with their spouse. Typical health problems were arthritis/rheumatism (67%), circulatory and blood-related disorders (62%), vision (48%), foot (45%) and hearing (42%). Half of the subjects were grouped to "high" mobility included 5 using walking aid. The other half of the subjects with "low" mobility required personal assistance. | ? | ? | ? | No results reported |
|  | English (US) | 35 |  | 72.86 ± 1.05 | 45.7% reported one or more falls within the last 12 months. | ? | ? | ? | ABC-16 may inflate overall confidence level reported by community-dwelling older adults potentially resulting in overestimation of true balance confidence |
|  | Turkish (Turkey) | 30 |  |  |  | Doubtful | / | / | No reported problems |
|  | Chinese-Cantonese (China HK) | 100 | 57 | 71.6 ± 23.7 | 17% use walking aids indoor, 37% use walking aids outdoors, 10% need assistance to walk outdoor, 13% has a fall history in past 6 months. 14% exercise less than 3 times per week | Inadequate | Inadequate | ? | For item 4, verbal explanation and additional demonstration of the action were needed for some respondents For item 10 (walking across a parking lot to the mall), the level of difficulty associated with performing such activity may have multiple interpretations by different respondents because of the different geographic locations in Hong Kong. |
|  | Mizo-tawng (India) | 20 |  |  |  | ? | ? | ? | No results reported |
|  | Hindi (India) | 403 | 48.64 | >60 |  | Inadequate | ? | ? | Respondents were not able to identify an escalator. Scale revised to a total of 14 items by removing item 14 and 15. |
|  | English (UK) | 30 |  |  | Patients attending a falls and syncopy facility | Doubtful | Doubtful | / | No further modification was required |
| CONFBal | English (UK) | 153 |  | 81 ± 6 | 57% required the use of a walking aid. 57% lived alone. 44% experience a fall in the last year. | ? | ? | ? | No results reported |
| **List of scales not measuring falls efficacy or balance confidence** | | | | | | | | | |
| Icon-FES | English (Australia) | 250 | 53.2 | 80.2 ± 5.1 | 36% reported one or more falls in the previous year | ? | ? | ? | No results reported |
| FES-I | English (UK) | 704 | 72.9 | 74.7 ± 7.1 | 53.4% experience one or more falls in the past year | ? | ? | ? | No results reported |
| MES | English (US) | 92 | 100 | 76.12 ± 6.75 |  | ? | ? | ? | No results reported |

**Foonotes**

RE: Relevance. CB: Comprehensibility. CV: Comprehensiveness. PT: Physiotherapy. FES: Falls Efficacy Scale. MFES: Modified Falls Efficacy Scale. PAPMFR: Perceived Ability to Prevent and Manage Fall Risks. GES: Gait Efficacy Scale. PCOF: Perceived Control Over Falling. PAMF: Perceived Ability to Manage Risk of Falls or Actual Falls. BSPT: Balance Self-Perception Test. ABC: Activities-specific Balance Confidence. CONFBal: CONFBal scale of balance confidence. Icon-FES: Iconographical Falls Efficacy Scale. FES-I: Falls Efficacy Scale-International. MES: Mobility Efficacy Scale. HLGD: High Level Gait Disorders. PD: Parkinson’s Disease. /: not applicable. ?: unclear if this content validity aspect was evaluated.
