# Supplementary material for: Falls efficacy instruments for community-dwelling older adults: a COSMIN-based systematic review
Source: BMC Geriatr. 2021 Jan 7;21:21. doi: 10.1186/s12877-020-01960-7 (PMC7792090; doi:10.1186/s12877-020-01960-7)
Supplement: Supplementary file 5 — Additional file 5. Characteristics, quality assessment and results of the content validity studies involving professionals. A table detailing information about the content validity studies involving professionals. [file 12877_2020_1960_MOESM5_ESM.docx]

**Additional file 5: Characteristics, quality assessment and results of the content validity studies involving professionals**

| **No** | **Name abbreviation** | **Reference** | **Language**  **(Country)** | **N** | **Profession(s)** | **RE** | **CV** | **Remarks** |
| --- | --- | --- | --- | --- | --- | --- | --- | --- |
| **List of falls efficacy scales** | | | | | | | | |
| 1 | FES-10 | Tinetti 1990 | English (US) | 20 | Physical therapists, occupational therapists, rehabilitation nurses and physicians | Doubtful | / | General consensus among professionals supported the face validity of the items with recommendation of more specific wordings for some items. |
|  |  | Buchner (1993) | English (US) |  | 4 FICSIT committees involving multi-site collaborative international study | ? | ? | The measure was changed to a four-category scoring system because of older persons' difficulty with the 10 levels of response categories. Wording was changed from "how confident" to "how concerned" because negative wording of the question may increase the spread of scores. |
| 2 | MFES-13 | Mosallanezhad 2011 | Persian (Iran) | 10 | Physiotherapists, physicians, occupational therapists and nurses | Doubtful | / | No difficulty in perception of the items and general instructions. |
| 3 | MFES-14 | French (France) | Perrot 2018 | 3 | Native French speakers familiar with the concept of fear of falling, English speaking translator | Doubtful | / | Minor revision e.g. "going up or downstairs" rather than "using front or rear steps at home" |
| **List of balance confidence scales** | | | | | | | | |
| 4 | ABC-15 | Filiatrault 2007 | English (Canada) | 5 | Rehabilitation and physical activity professionals and researchers | Doubtful | / | Minor changes in word usage e.g. 'mall' was replaced to 'shopping center' |
| 5 | ABC-16 | Qiang Guan 2012 | Chinese-Mandarin (China) | 3 | Professional researchers | Doubtful | / | Three major changes of the original items were made:  (1) item 3 (bending over to pick up a slipper from the floor in front of a closet) was changed to item 3 (bending over to pick up a slipper from the ground)  (2) item 8 (walking outside the house to a car parked in the driveway) was changed to item 8 (taking a taxi)  (3) item 9 (getting into or out of a car) was changed to item 9 (taking and getting down from the bus) |
|  |  | Arnadottir 2010 | Icelandic (Iceland) | 4 | 3 physiotherapists and 1 language specialist | Doubtful | / | Some of the test items required an extra discussion because there are no escalators or malls in rural areas os Iceland. However, these items were retained as respondents are asked to imagine how confident they are when doing the activity.  The word "mall" was translated to Icelandic as a word with a broader meaning to various clusters of shops such as malls, strip malls, shopping centers. |
|  |  | Marques 2013 | Brazilian-Portugese (Brazil) | 7 | Two rheumatologists, one orthopaedic surgeon, two PT researchers and 2 PT students | Doubtful | / | Six of the original items were modified.  Item 3 (bending over to pick up a slipper from the floor in front of a closet) was replaced to item 3 (stooping to pick up an object on the ground); Item 8 “walk outside the house to a car parked in the driveway” was changed to “walk outside the house to a car or bus stopped in front”; Item 10, “walk across a parking lot to the mall” was changed to “walk across a parking lot of a supermarket or a mall”; Item 12 and 13, the word ‘”mall” was replaced  by “crowded place”; Item 16, “walk outside on icy sidewalks” was changed to “walk outside on slippery sidewalk” |
|  |  | Ayhan 2014 | Turkish (Turkey) |  | Translators, academics and interested professionals | Doubtful | / | 3 items were modified. For item 4, "height level" was replaced with "eye level". For item 8, "to a car parked in a driveway" was modified as "to the street". For item 16, "icy sideways" was replaced with "slippery sidewalks" |
|  |  | Mak 2007 | Chinese-Cantonese (China HK) | 10 | Physiotherapists | Doubtful | / | 4 items were judged "culturally irrelevant":  (1) item 3 bending over to pick up a slipper from the floor in front of a closet) was changed to item 3 (bending over to pick up a slipper from the floor)  (2) for item 8 and 9, "car" was replaced with "transportation"  (3) for item 16, "icy sidewalks" was replaced with "wet and slippery pavement"  Slight modifications to the original testing instructions were tailored to elderly respondents who were less educated. |
|  |  | Ralte 2017 | Mizo-tawng (India) |  | Medical specialists, senior medical officer, physiotherapists | Doubtful | / | 9 items were modified. For item 3, "slipper" was changed to an "object", "wearing shoe by yourself". For item 4, "small can" was changed to "medicine bottle". Item 10 was modified to "walk across a busy traffic road". For item 11, "ramp" was changed to "slope road". In items 12 and 13, the word "mall" was replaced with "crowded market". In item 14 and 15, "escalator" was changed to "unsteady stairs". For item 16, "icy" was replaced with "slippery" |
|  |  | Parry 2001 | English (UK) |  | Investigators and health services researchers | Doubtful | / | Unfamiliar words such as "sidewalk", "mall" and "closet" were changed to "pavement", shopping centre" and "cupboard" respectively. |
| **List of scales not measuring falls efficacy or balance confidence** | | | | | | | | |
| 6 | Icon-FES | Delbaere 2011 | English (Australia) | 4 | Falls-related experts | / | Doubtful | Two additional items were added to the original list of 30 items |

**Foonotes**

RE: Relevance. CV: Comprehensiveness. PT: Physiotherapy. FES: Falls Efficacy Scale. MFES: Modified Falls Efficacy Scale. ABC: Activities-specific Balance Confidence. Icon-FES: Iconographical Falls Efficacy Scale. /: not applicable. ?: unclear if this content validity aspect was evaluated.
