# Supplementary material for: Falls efficacy instruments for community-dwelling older adults: a COSMIN-based systematic review
Source: BMC Geriatr. 2021 Jan 7;21:21. doi: 10.1186/s12877-020-01960-7 (PMC7792090; doi:10.1186/s12877-020-01960-7)
Supplement: Supplementary file 6 — Additional file 6. Characteristics, quality assessment, and results of the structural validity studies of instruments measuring falls-related self-efficacy or balance confidence in community-dwelling older adults. A table detailing information about the structural validity studies of instruments measuring falls-related self-efficacy or balance confidence in community-dwelling older adults. [file 12877_2020_1960_MOESM6_ESM.docx]

**Additional file 6: Characteristics, quality assessment, and results of the structural validity studies of instruments measuring falls-related self-efficacy or balance confidence in community-dwelling older adults**

| **Name abbreviation** | **Reference** | **Country (language)** | **N** | **Female (%)** | **Age** | **Need aids (%)** | **History of 1 or more falls** | **Scale score** | **COSMIN quality rating** | **Analysis model** | **Results** |
| --- | --- | --- | --- | --- | --- | --- | --- | --- | --- | --- | --- |
| **List of falls efficacy scales** | | | | | | | | | | | |
| FES-10 | Hill, 1996 | English (Australia) | 179 | 79.9 | 79.2 ± 6.6 (n=68 with fall)  74 ± 4.1 (n=111 no fall) |  | 38% in the past year |  | Very good | PAF with eigenvalues > 1 | One factor accounting for 69% of the sample variance |
| MFES-14 | Perrot, 2018 | France (French) | 310 | 64.2 | 74.7 ± 8.7 |  |  |  | Adequate | EFA extracting factors with eigenvalues >1 with OFR | Two factors accounting for 68.1% of total variance.  A single factor could account 61.7% of the variance. |
|  | Hill, 1996 | English (Australia) | 179 | 79.9 | 79.2 ± 6.6 (n=68 with fall)  74 ± 4.1 (n=111 no fall) |  | 38% in the past year | 7.69 ±2.21  9.76 ± 0.32 | Very good | PAF with eigenvalues > 1 | Two factors accounting for 75% of total variance |
| PAPMFR | Yoshikawa, 2019 | English (US) | 522 | 82.4 | 76.45 ± 7.79 |  | 18% in the past month | 3.33 ± 0.91 (those without a fall)  2.47 ± 0.82 (those had one or more falls) | Very good | EFA with CFA | EFA: one factor with eigenvalue > 1  CFA: good fit 1-factor model with correlated items  (RMSEA = 0.03, CFI = 1.00, WRMR = 0.23). |
| **List of balance confidence scales** | | | | | | | | | | | |
| ABC-6 | Schott, 2014 | Germany (German) | 384 | 57.3 | 71.1 ± 9.7 |  | 35.2% in the past year | 74.1 ± 27.1 | Very good | PCA with VR | One factor accounted for 79.8% of total variance |
| ABC-15 | Filiatrault, 2007 | Canada (English) | 197 | 84 | 73.9 ± 7.4 |  | 38% in the past year | 6.4 ± 2.1 | Very good | Polytomous IRT (SGRM) | Two factors accounted for 27% of the variance. |
| ABC-16 | Qiang Guan, 2011 | China  (Chinese-Mandarin) | 61 | 57 | 76.3 ± 7.5 | 16 | 21% in the past 6 months | 74.6 ± 12.4 | Inadequate | PCA with VR | Two factors accounted for 52.5% and 13.6% respectively |
|  | Arnadotti, 2010 | Iceland (Icelandic) | 183 | 47.5 | 73.8 ± 6.2 | 13.7 | 31.6% in the past year | 83.3 ± 18.3 | Adequate | Rasch (model not specified) | Three items failed to show acceptable levels of fit.  (Item 7 “sweep the floor”, infit mean square = 1.45, outfit mean square = 1.96; Item 3 “pick up a slipper”, infit mean square = 1.81, outfit mean square = 1.77; Item 15 “on/off escalator while holding parcels”, infit mean square = 1.40, outfit mean square = 1.24) |
|  | Wang, 2018 | English (US) | 5012 | 65 | 73.6 ± 5.8 |  |  |  | Very good | Rasch PCM  EFA and CFA | Two items (item 16 and item 4) were noted for marginal misfit, where fit statistics were greater than 1.40.  First factor accounted for 77% of the variance, second 6% and third 3% |
|  | Schott, 2014 | Germany (German) | 384 | 57.3 | 71.1 ± 9.7 |  | 35.2% in the past year | 83.4 ± 21.7 | Very good | PCA with VR | One factor accounted for 72.6% of total variance |
|  | Ayhan, 2014 | Turkey (Turkish) | 106 | 46 | 69.52 ± 5.17 |  | 54.7% in the past year | 86.97 ± 22 | Adequate | PCA with VR | Two factors accounted for 68.651% and 9.978% respectively of the variance |
|  | Mak, 2007 | China HK (Chinese-Cantonese) | 100 | 57 | 71.6 ± 23.7 | 37 | 13% in the past 6 months | 71.6 ± 23.7 | Adequate | PCA with VR on factors with eigenvalues > 1 | One factor accounted for 69.66% of total variance |
| **List of scales not measuring falls efficacy or balance confidence** | | | | | | | | | | | |
| Icon-FES | Delbaere, 2011 | English (Australia) | 250 | 53.2 | 80.2 ± 5.1 |  | 36% in the past year | 48.75 ± 14.65 (those without a fall)  58.02 ± 19.46 (those had one or more falls) | Very good | Rasch (model not specified)  PCA with VR | Excellent overall fit for most items except for "Catching the bus when you have to stand" which the item was then deleted.  Two factors accounted for 29.43% and 23.25% of the variance respectively. Single factor able to explain 45.6% of the variance. |
| FES-I | Yardley, 2005 | English (UK) | 704 | 72.9 | 74.7 ± 7.1 |  | 53.4% in the past year |  | Very good | PCA with VR | Two factors accounted for 36.8% and 32.7% of the variance respectively.  A single factor could account 61.7% of the variance |

**Footnotes**

PAF: Principle Axis Factoring. EFA: Exploratory Factor Analysis. OFR: Oblique Factor Rotation. CFA: Confirmatory Factor Analysis. RMSEA: Root Mean Square Error of Approximation. CFI: Confirmatory Fit Index. WRMR: Weighted Root Mean Square Residual. PCA: Principal Component Analysis. VR: varimax rotation. IRT: Item Response Theory. SGRM: Samejima’s graded response model. PCM: Partial Credit Model.
