# Supplementary material for: Falls efficacy instruments for community-dwelling older adults: a COSMIN-based systematic review
Source: BMC Geriatr. 2021 Jan 7;21:21. doi: 10.1186/s12877-020-01960-7 (PMC7792090; doi:10.1186/s12877-020-01960-7)
Supplement: Supplementary file 7 — Additional file 7. Evidence synthesis on the content and structural validity of instruments measuring falls-related self-efficacy or balance confidence in community-dwelling older adults. A table detailing the synthesis of evidence on the content validity and structural validity of falls-related self-efficacy instruments or balance confidence instruments for the community-dwelling older adults. [file 12877_2020_1960_MOESM7_ESM.docx]

**Additional file 7: Evidence synthesis on the content and structural validity of instruments measuring falls-related self-efficacy or balance confidence in community-dwelling older adults**

| **Name abbreviation** | **Relevance rating of results** | **Relevance quality of evidence** | **Comprehensiveness rating of results** | **Comprehensiveness quality of evidence** | **Comprehensibility rating of results** | | **Comprehensibility quality of evidence** | **SV rating of results** | **SV quality of evidence** | |
| --- | --- | --- | --- | --- | --- | --- | --- | --- | --- | --- |
| **List of falls efficacy scales** | | | | | | | | | | |
| FES-10 | + | Moderate | - | Very low | + | | Very low | + | High | |
| MFES-11 | + | Very low | - | Very low | - | | Very low | ? | ? | |
| MFES-12 | + | Very low | - | Very low | - | | Very low | ? | ? | |
| MFES-13 | + | High | - | High | ± | | Moderate | ? | ? | |
| MFES-14 | + | Moderate | - | Very low | + | | Moderate | + | High | |
| PAPMFR | + | Very low | - | Very low | + | | Very low | + | High | |
| GES-8 | + | Very low | - | Very low | + | | Very low | ? | ? | |
| GES-10 | + | Very low | - | Very low | + | | Very low | ? | ? | |
| PCOF | + | Very low | - | Very low | + | | Very low | ? | ? | |
| PAMF | + | Very low | - | Very low | + | | Very low | ? | ? | |
| BSPT | + | Very low | - | Very low | + | | Very low | ? | ? | |
| **List of balance confidence scales** | | | | | | | | | | |
| ABC-6 | - | Very low | - | Very low | + | | Very low | + | High | |
| ABC-15 | + | Moderate | - | Very low | + | | Very low | + | High | |
| ABC-16 | + | Low | + | Low | ± | | Low | + | High | |
| CONFBal | + | Low | + | Low | + | | Low | ? | ? | |
| **List of scales not measuring falls efficacy or balance confidence** | | | | | | | | | | |
| Icon-FES | + | Moderate | + | Moderate | | + | Low | + | | High |
| FES-I | + | Very low | - | Very low | | + | Very low | + | | High |
| MES | + | Low | + | Low | | + | Very low | ? | | ? |

**Footnotes**

Ratings: Sufficient (+); Insufficient (-), Inconsistent (±), Indeterminate (?) are based on standards described in the COSMIN guidelines. More details listed in the article published by Terwee CB, Prinsen CA, Chiarotto A, De Vet HC, Westerman MJ, Patrick DL, et al. . COSMIN Methodology for Evaluating the Content Validity of Health-Related Patient-Reported Outcome Measures: A Delphi study. Qual Life Res. 2018;27(5):1159-1170
